# Epitope-Specific Anti-hCG Vaccines on a Virus Like Particle Platform

**DOI:** 10.1371/journal.pone.0141407

**Published:** 2015-10-30

**Authors:** Jerri Caldeira, Jeremiah Bustos, Julianne Peabody, Bryce Chackerian, David S. Peabody

**Affiliations:** Department of Molecular Genetics and Microbiology, University of New Mexico School of Medicine, Albuquerque, New Mexico 87131, United States of America; Aaron Diamond AIDS Research Center with the Rockefeller University, UNITED STATES

## Abstract

The possibility of a contraceptive vaccine targeting human chorionic gonadotropin has long been recognized, but never fully realized. Here we describe an epitope-specific approach based on immunogenic display of hCG-derived peptides on virus-like particles of RNA bacteriophage. A number of recombinant VLPs were constructed, each displaying a different hCG-derived peptide. Some were taken from the disordered C-terminal tail of the hormone, another came from an internal loop, and yet another was an epitope mimic produced by affinity-selection on an hCG-neutralizing antibody target. Immunization of mice with some VLPs yielded antisera that bound the hormone and neutralized hCG biological activity.

## Introduction

Efforts to produce a contraceptive vaccine targeting human chorionic gonadotropin (hCG) face several important challenges. First, the vaccine must avoid eliciting antibodies that cross-react with the closely related luteinizing, follicle-stimulating, and thyroid-stimulating hormones (LH, FSH and TSH). Second, the vaccine has to strike the difficult balance of provoking an antibody response of sufficient duration to be useful, without causing life-long infertility. That is, it must be effective, but ultimately reversible. Third, in humans hCG is a self-antigen so the vaccine must be sufficiently immunogenic to overcome B-cell tolerance.

Although their activities differ, the hormones hCG, LH, FSH and TSH are structurally similar. Each contains an identical 92-amino acid α-chain and are therefore distinguished only by differences in their β-chains, and even there significant sequence homology raises the possibility of immunologic cross-reaction. However, the hCG ß-chain possesses a C-terminal tail that is absent from the other proteins [1, 2], and therefore should serve as a source of potential vaccine epitopes unique to hCG. The full length of the 30-amino acid tail has been used previously as an immunogen in animals, where it elicited hCG neutralizing antibodies without incurring LH cross-reactivity [3]. However, the anti-hCG titers were far lower than when the entire hCG molecule was used as immunogen [4]. Additionally, repeated immunizations with strong adjuvants were required [5].

The multivalent and nano-particulate nature of virus-like particles (VLPs) makes them highly immunogenic scaffolds for display of diverse epitopes [6–8]. In fact, they are immunogenic enough to overcome B-cell tolerance and elicit antibodies against self-antigens like hCG. VLPs produce long-lived, high-titer antibody responses at low doses even in the absence of adjuvants. We previously described the development of a VLP platform based on the coat proteins of the RNA bacteriophages MS2 and PP7, which facilitates immunogenic display of peptide epitopes [9, 10]. It depends on single-chain dimer versions of the MS2 and PP7 coat proteins, which we specifically engineered to tolerate diverse peptide insertions in a surface loop [11, 12]. When expressed in *E*. *coli*, recombinant coat proteins containing such insertions self-assemble into VLPs that display the foreign peptide on their surface.

Not only does the MS2 VLP serve as a platform for display of specific peptides, it can also be used for affinity-selection of peptide epitope mimics (mimitopes) in a process similar to phage display. Briefly, complex random sequence peptide libraries are constructed in the coat protein AB-loop. These libraries are subjected to affinity-selection (biopanning) on an antibody target, and because the VLP encapsidates its own mRNA, bound sequences can be recovered by reverse transcription and polymerase chain reaction. Expressing the recovered sequences in *E*. *coli* produces VLPs that now display only peptides that bind the selecting antibody [10, 12]. This results in the identification of peptides that mimic the antibody’s epitope and that can often elicit antibodies of the same specificity.

Summarizing, the RNA phage VLP enables the production of vaccine candidates by two different routes. First, we can introduce known peptide epitopes into the coat protein surface loop and use them directly as vaccine antigens, and second, we can identify the epitope (or an epitope mimic) by affinity selection. Immunization with the affinity selected VLP often elicits antibodies that recognize the original antigen. We utilized both these approaches attempting to produce VLPs to induce antibodies that neutralize hCG.

## Materials and Methods

### Plasmids and proteins

Peptides derived from several locations in the hCG sequence were inserted genetically into the AB-loop of PP7 coat protein by methods described previously using the plasmid we call p2P7K32 [9]. To confirm whether a given construct produced a coat protein able to properly fold and assemble, we determined the presence or absence of an intact VLP by electrophoresis of cell lysates on 1% agarose gels, and by size exclusion chromatography on Sepharose CL-4B [12].

### Affinity selection

The details of affinity selection by biopanning in the MS2 VLP system were briefly described in the introduction to this paper and extensively in reference [10]. We used a mixture of 6-mer, 7-mer 8-mer and 10-mer random sequence peptide libraries, each of which contained about 10^10^ individual recombinants. A total of four selection rounds were conducted, the first two at high peptide display valency (in pDSP62), and the last two at low valency (in pDSP62(am)) to increase selection stringency [10]. The products of the final round were characterized by DNA sequence analysis. The selected peptide was re-cloned into pDSP62 for display at high valency and VLPs were purified as described before [10] for use in immunizations.

### Immunizations and ELISA

Mice were immunized intramuscularly three times at two week intervals with 5μg of VLPs plus incomplete Freund’s Adjuvant (IFA) in a total volume of 100 μl. Antibody responses were characterized by ELISA using standard methods.

### Bioassay of the hCG-neutralizing capacity of the sera

The bioactivity of hCG was quantified by comparing the weights of the uterus of immature female mice after hCG treatment [13]. In each of two independent experiments, twenty-seven immature C57BL/6 females (20 days old, from Harlan Laboratories, Inc.) were randomly divided into 9 groups (three mice per group). Animals received three subcutaneous hCG injections, one on each of three consecutive days at the same time-of-day. Each injection consisted of 100μl of PBS containing 200 ng of hCG. To test for hCG neutralization, some samples contained filter sterilized anti-VLP sera at a 1:1 dilution. All samples were incubated for 30 min at 37°C before injection to allow reaction of antibody with the hormone. On the fourth day (i.e. 24 hours after the third hCG injection) mice were sacrificed and the uterus was removed, trimmed and weighed. The measurements are presented as the mean±SEM of the uterine mass (in mg) per 100 grams of body weight. Statistical analysis was performed using Student’s t-test, and *p<0*.*05* was considered significantly different from the corresponding control.

### Animal care

All animal care was conducted according the National Institutes of Health and University of New Mexico guidelines. The experiments reported here were specifically approved by the University of New Mexico Institutional Animal Care and Use Committee. See S1 Text.

## Results

### Display of hCG-specific peptides on PP7 VLPs

The RNA phage coat proteins possess a prominent surface loop (the AB-loop) where foreign peptides can be displayed in an immunogenic fashion. A specially engineered “single-chain dimer”, created by genetically joining the two dimer halves into a single polypeptide chain, is highly tolerant of loop insertions [9, 10]. Accordingly, we constructed a series of PP7 VLPs displaying the hCG-derived peptides shown in Table 1. Some were derived from the unique C-terminal tail of the hCG ß-chain. They were constructed as serial 10-mers scanning through the sequence with 5-amino acid overlaps, and included residues 111–120, 116–125, 121–130, 131–140 and 136–145. Other peptides were derived from internal loops in the hCG ß-chain.

**Table 1:** Sequences of the hCG-derived peptides displayed on the VLP.

39-PTMTRVLQGVLPALPQVV-56
45-LQGVLPALPQV-55
66-SIRLPGCPRGVNPVVS-81
69-LPGCPRGVNPV-80
111-DDPRFQDSSS-1
116-QDSSSSKAPP-125
121-SKAPPPSLPS-130
126-PSLPSPSRLP-135
131-PSRLPGPSDT-140
136-GPSDTPILPQ-145
5AS-ERQFGRKSGR

Oligonucleotides encoding the peptide sequences shown here were inserted into the PP7 coat protein AB-loop. The numbers represent the positions of the starting and ending amino acids of each peptide in the hCG sequence.

**Fig 1:**
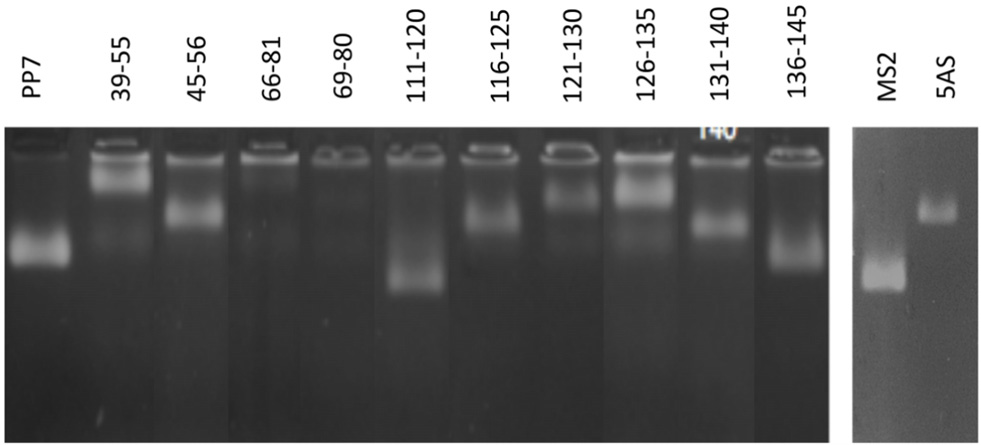
Agarose gel electrophoresis of the wild type and the recombinant VLPs. The particles contain RNA and are therefore visualized by staining with ethidium bromide. The differences in mobility are consequences of alterations in VLP surface charge due to the presence the foreign peptides. Each peptide is identified with numbers corresponding to the amino acid residues at its N- and C-terminal boundaries in the hCGß sequence. 5AS refers to the cPiPP antibody affinity selectant (see text for details).

Out of the ten peptide insertions we constructed, eight yielded recombinant coat proteins that folded and assembled correctly as judged by agarose gel electrophoresis of the VLPs (Fig 1). Since each particle contains RNA, it is easily visualized by ethidium bromide staining. Each VLP has a distinct mobility determined by the surface charge of the PP7 VLP itself and by any charges added by the foreign peptide. A western blot of the agarose gel probed with anti-PP7 serum shows that the ethidium-staining band co-migrates with PP7 coat protein (not shown). Furthermore, each VLP behaves like the unaltered VLP during purification by size exclusion chromatography in Sepharose CL-4B, also demonstrating that each protein assembled into a VLP-sized particle (not shown). Unfortunately, we were unable to obtain usable quantities of the VLP with the peptide we call 45–55. Although, as Fig 1 shows, we initially encountered normal amounts of the VLP in cell lysates, it is apparently unstable over the longer time frames required for purification and was lost. This left us with seven stable peptide-displaying PP7 VLPs for immunization tests.

### Affinity-selection of an hCG peptide epitope mimic

Above we described the production of PP7 VLPs displaying known peptides derived directly from the amino acid sequence of hCG. But the RNA phage VLP system also allows discovery of epitopes and epitope mimics through an affinity selection process similar to phage display. Elsewhere we described in detail the development of this peptide display and affinity-selection platform based on the RNA phage called MS2 [9, 10, 12]. PP7 and MS2 are highly similar phages, but of the two we have more thoroughly characterized the MS2 VLP as an affinity selection platform and therefore used it for these experiments. We construct random-sequence peptide libraries on MS2 VLPs and routinely affinity-select peptide epitopes by biopanning on antibody targets with the goal that the resulting VLPs will elicit antibodies whose activities mimic those of the selecting antibodies (reference [10] and unpublished results). In this case, the target antibody was the monoclonal called cPiPP (the kind gift of GP Talwar), an antibody that neutralizes the activity of hCG by interaction with an unmapped, but presumably conformational epitope [14]. After four rounds of affinity-selection on cPiPP, sequence analysis identified a VLP displaying the sequence ERQFGRKSGR. We call this VLP 5AS. The peptide shows no obvious homology to any peptide in hCG, consistent with the possibility that it mimics a non-linear epitope.

### Immunizations and Analysis of Antisera

**Fig 2:**
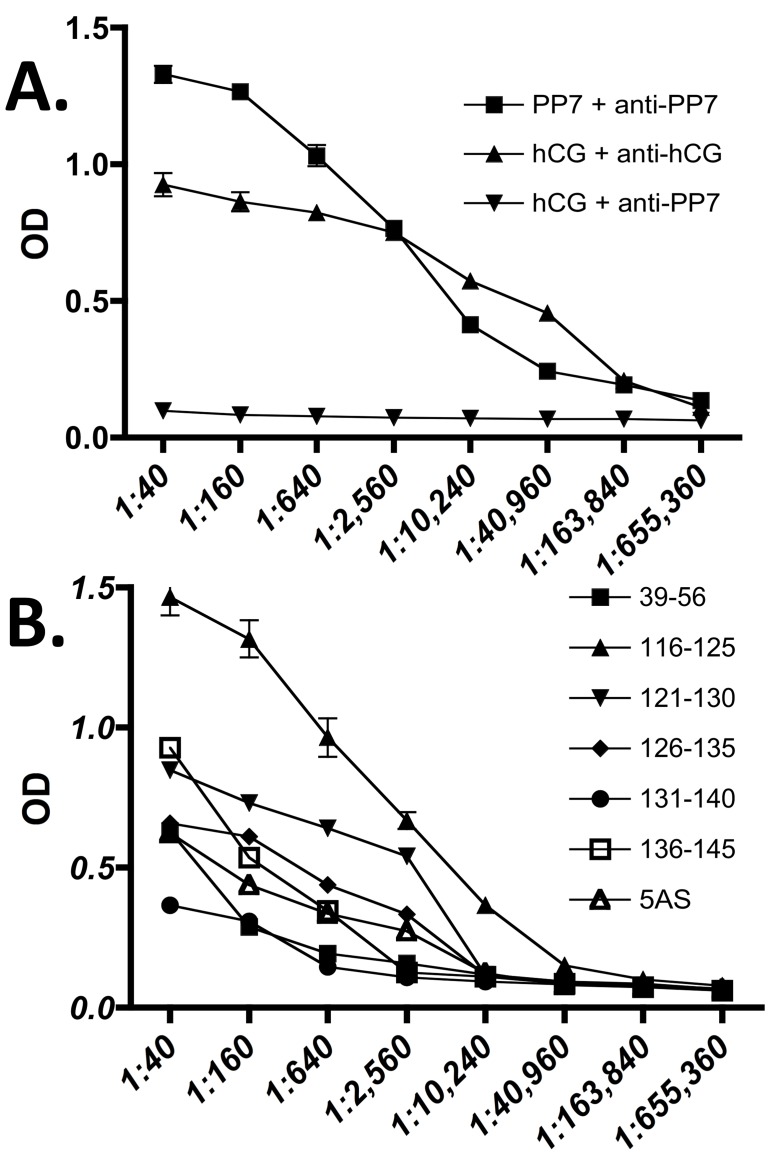
ELISA of anti-sera from mice immunized with VLPs displaying the various peptides described in the text. As controls **panel A** shows that anti-PP7 serum reacts with the PP7 VLP but not with hCG. Also shown is reaction of an anti-hCG monoclonal antibody with hCG. **Panel B** shows reaction with hCG of antisera generated by immunization of mice with each of the indicated peptide-displaying VLPs. In each case, three mice were immunized, and the values plotted here are the averages.

Both the PP7- and MS2-derived VLPs described above were synthesized in *E*. *coli* and purified by methods we described before [12, 15]. Mice were immunized and their sera were tested for reaction with hCG by ELISA (see Materials and Methods). We note that although we utilized incomplete Freund’s adjuvant in these experiments, VLPs do not depend on such an adjuvant to provoke a strong antibody response [16]. Although there was some variation in the apparent titers obtained with different VLPs, all produced a substantial anti-hCG response (Fig 2). Some peptides (116–125, 126–135 and 136–145) produced stronger ELISA signals than others, but it is unclear whether this reflects intrinsic differences in immunogenicity, or differential accessibility of epitopes on hCG immobilized on a plastic surface.

**Fig 3:**
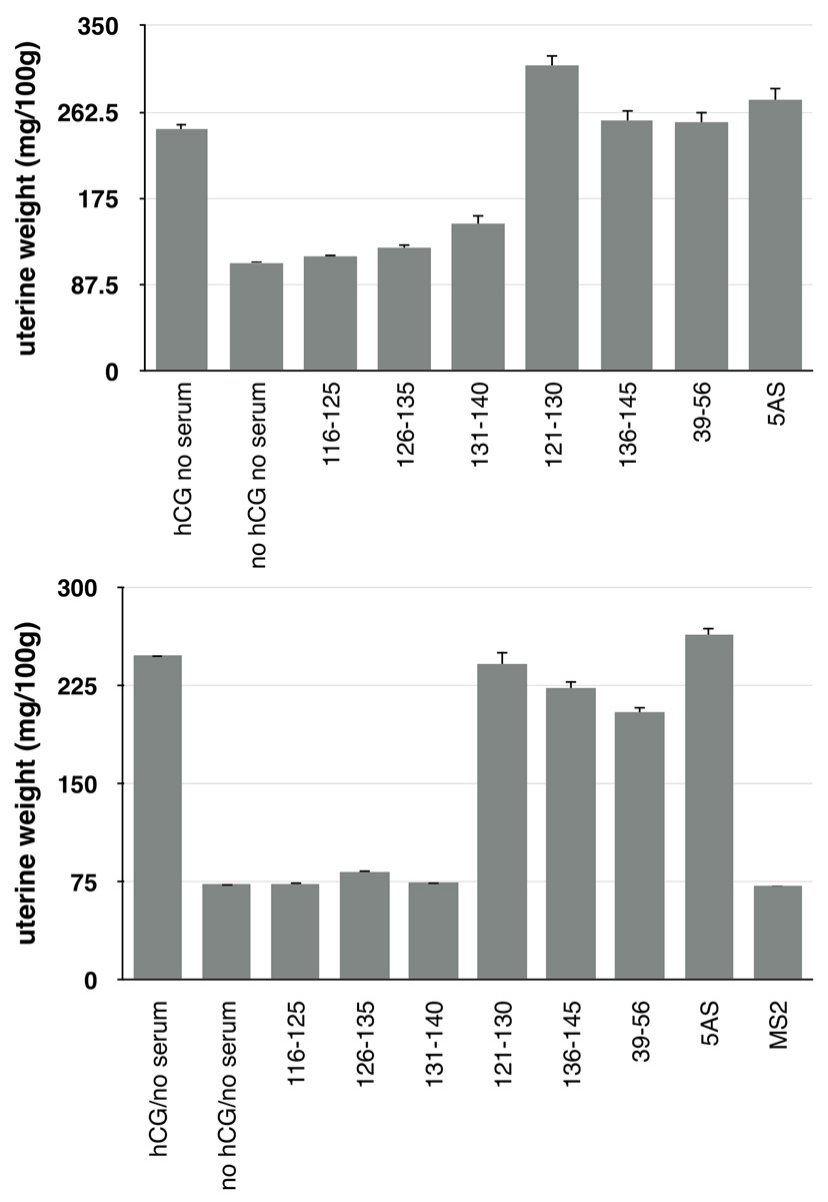
Neutralization of hCG bioactivity by anti-hCG serum. The upper and lower panels show the results of two independent experiments in which immature mice were injected for three consecutive days with 100μl of PBS containing 200ng of hCG plus sera from mice immunized with the various VLPs identified by the peptides they display (see the text). Also shown are control experiments without serum (no serum), with neither serum nor hormone (no serum no hormone) and with anti-MS2 serum (MS2, present only in the lower panel). The results are presented as mg of uterine weight per 100 grams body weight.

Although mice lack chorionic gonadptropin, when hCG is injected into immature mice it causes a rapid growth of the reproductive organs, thus providing a simple bioassay for antibody neutralization [13]. When we treated immature mice with hCG alone, or with the hormone incubated with control serum from mice immunized with unmodified PP7 VLPs (*p<0*.*05*), uterine weight nearly doubled over the course of three days (Fig 3). Preincubating the hormone with antiserum elicited by VLPs displaying peptides 121–130 and 136–145 also failed to inhibit hCG activity. However, sera generated by immunization with VLPs displaying peptides 116–125, 126–135 and 131–140 dramatically inhibited uterine weight gain. Note that each result presented in Fig 3 represents the average of three individual animals, and the entire experiment was conducted twice on separate occasions to ensure reproducibility.

## Discussion

A contraceptive vaccine that is safe, long-lasting, yet reversible, and which lacks collateral effects on homologous hormones presents significant challenges. Several vaccine targets have been tested to date, but none has so far satisfied all these requirements. For example, antigens of the zona pellucida have been targeted because of their key role in the initial stages of fertilization, but their use has been associated with undesirable cross reactions resulting in loss of primordial follicles from the ovary and reduced ovarian function [17]. Sperm surface antigens have also been considered, but progress has been limited because of the presence of antigens shared with somatic cells [18, 19]. Gonadotropin Releasing Hormone (GnRH) exhibits some attractive features, including its presence in both sexes, which suggests the possibility of a contraceptive vaccine active in males and females. Unfortunately, because of GnRH’s role in sex steroid production, fertility, and libido, the inevitable side-effects of such a vaccine would likely make it unacceptable for human use, although it could have utility in animals.

So far the most successful efforts to produce a contraceptive vaccine for humans have focused on hCG. The hormone is produced only after fertilization, and since it is required for implantation, inhibiting its activity prevents pregnancy. As an auto-antigen, hCG is subject to B-cell tolerance, which makes it difficult to attain a high-titer antibody response. Nevertheless, Talwar and co-workers produced a vaccine by attaching hCG's ß-subunit to tetanus toxoid. Its safety and reversibility were tested in clinical trials through Phase I, where it was shown that vaccination did not alter menstrual regularity, the secretion of LH, TSH or FSH, nor the production of estrogen and P4 [20, 21]. Unfortunately, it failed to generate a strong enough antibody response in a large enough fraction of vaccinees to reliably prevent pregnancy. In a later attempt, the β-subunit of hCG was paired with the α-subunit of ovine LH to produce a heterospecies dimer, which was then linked to tetanus toxoid or to diphtheria toxoid and adsorbed on alum with the sodium pthalyl derivative of lipopolysacaride (SPLPS) as an adjuvant [22]. In Phase II trials the vaccine prevented pregnancy whenever anti-hCG titers exceeded about 50ng/ml. Vaccination did not alter the normal menstrual cycle, and the effect was reversible in the absence of boosting. However, only 80% of vaccinated women developed antibodies above the required 50ng/mL threshold, and this minimum level was sustained for 3 months or more in only 60%.

In the experiments described here we focused on an epitope-specific approach, using small hCG-derived peptides displayed on VLPs. To find epitopes unique to hCG, we concentrated on its unique C-terminal tail. Several prior studies utilizing the entire 36-amino acids of the tail were conducted in animal models, but with only limited success [23, 24], apparently due to relatively poor immunogenicity. For example, one study showed that peptides from the tail provoke neutralizing antibodies in rabbits [25]. There the immunogens differed significantly from ours; a series of B-cell epitopes from the CTP were linked to T-cell epitopes derived from tetanus toxoid and from Hepatitis B Virus and expressed as artificial proteins in *E*. *coli*. That study also measured the hCG-neutralizing activity of the sera using the uterine weight gain bioassay in immature mice. The results were similar to those we show in Fig 3, but four or five immunizations and strong adjuvants were required to obtain the observed antibody titers.

Our results confirm that an antibody response to peptides from the C-terminal tail can be neutralizing, but since we used relatively small peptides (10-mers), we can also roughly map the locations of neutralizing epitopes. The antisera produced against the peptide sequences between amino acids 116–125, 126–135 and 131–140 efficiently inhibited hCG activity. Interestingly, 121–130 and 136–145, which overlap the other three, apparently failed to provoke provoke neutralizing antibodies, even though each elicited antibodies that recognized the hormone in ELISA. Moreover, the strength of reaction of the various sera with hCG in ELISA correlates only imperfectly with their neutralization activities. This could be a result of differential accessibility of the various epitopes in hCG immobilized on a plastic surface compared with their accessibility in the molecule in solution, or it may reflect a true difference in the neutralizing potential of the individual epitopes, We don’t know the mechanism of neutralization, but given the distance of the C-terminal tail from hCG’s receptor-binding surface, it seems unlikely to be the result of direct inhibition of receptor binding.

Affinity selection on the MS2 VLP platform using the cPiPP antibody as a biopanning target yielded a peptide (ERQFGRKSGR) with no obvious homology to the hCG sequence itself, suggesting that it probably mimics a conformational epitope [26]. The fact that the 5AS-VLP elicits antibodies that recognize hCG in ELISA indicates that the peptide is an immunogenic mimic of the cPiPP epitope, but given cPiPP’s hCG-neutralizing activity, it is surprising that the anti-5AS-VLP serum fails to neutralize, especially since in ELISA it binds hCG as well, or better than some others that do show neutralizing activity. Mimicking a complex epitope with small peptide can be difficult, and ours probably mimics the cPiPP epitope imperfectly. Maybe it only mimics a portion of a larger epitope, which must be recognized in its entirety for neutralization to occur.

The immunogenicity of VLPs, their ability to break immune tolerance, and the relative durability [16] of the antibody responses they generate are favorable factors for future development of this and other vaccine candidates [27]. Obviously, it remains to be determined whether our VLP immunogens can elicit sufficiently high and appropriately durable antibody responses to hCG as a self-antigen in humans. Although here we concentrated on the possibility of a contraceptive vaccine, it is worth pointing to the growing number of studies that report the involvement of hCG in growth of certain cancers [28–30]. This raises the possibility of a vaccine that could have therapeutic efficacy for some tumors.

## Conclusions

Several different 10-amino acid peptides from the C-terminal tail of hCG displayed on the PP7 VLP are able to elicit antibodies in mice that efficiently inhibit hCG bioactivity. Since the peptides share no sequence similarity with LH, FSH, and TSH there is little chance of eliciting antibodies that cross-react with these hCG-related hormones.

## Supporting Information

S1 Text ARRIVE checklist. (DOCX)
