# Supplementary figures and images for: Epitope-Specific Anti-hCG Vaccines on a Virus Like Particle Platform
Source: PLoS One. 2015 Oct 30;10(10):e0141407. doi: 10.1371/journal.pone.0141407 (PMC4627648; doi:10.1371/journal.pone.0141407)

S1 Text. The ARRIVE Guidelines Checklist.


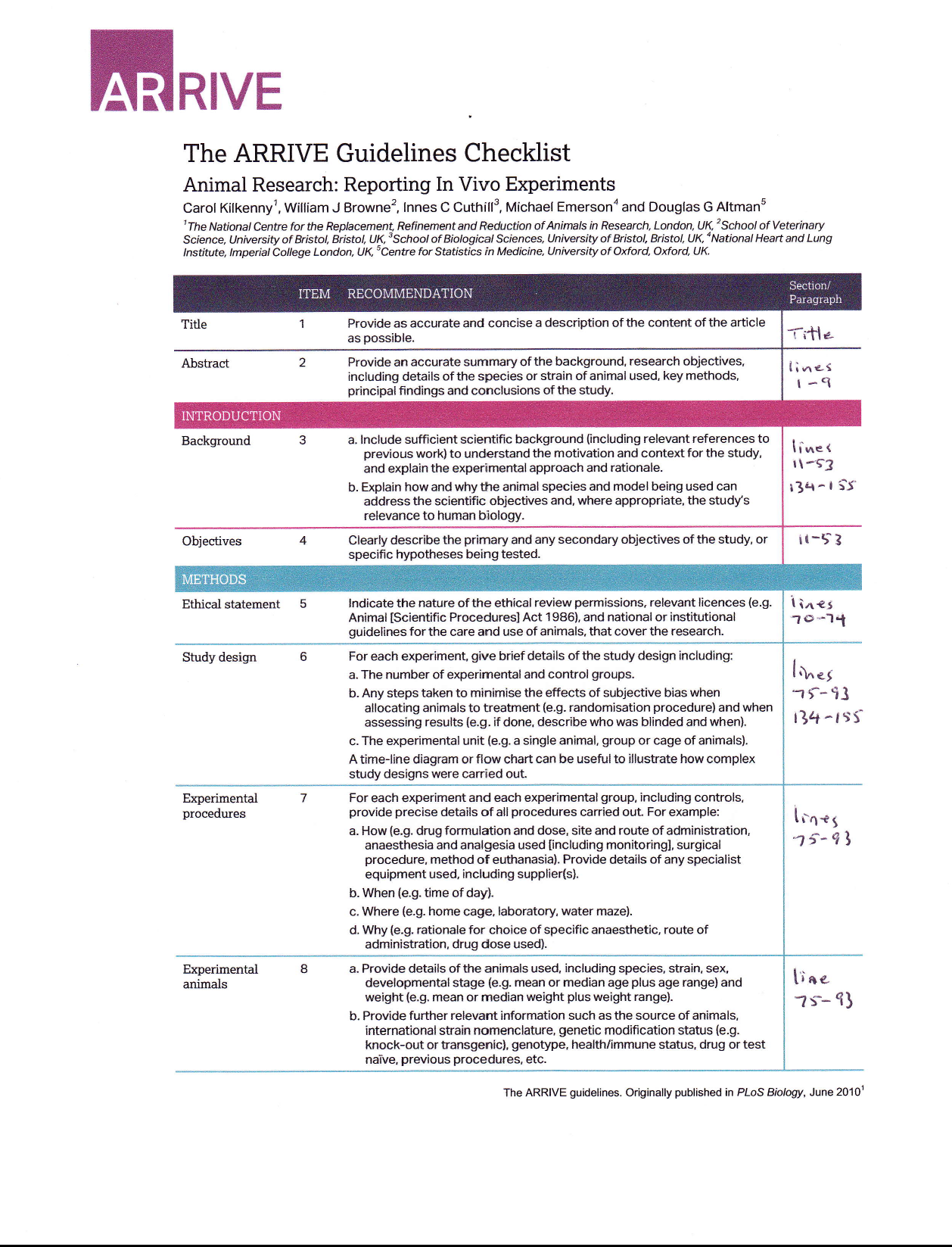


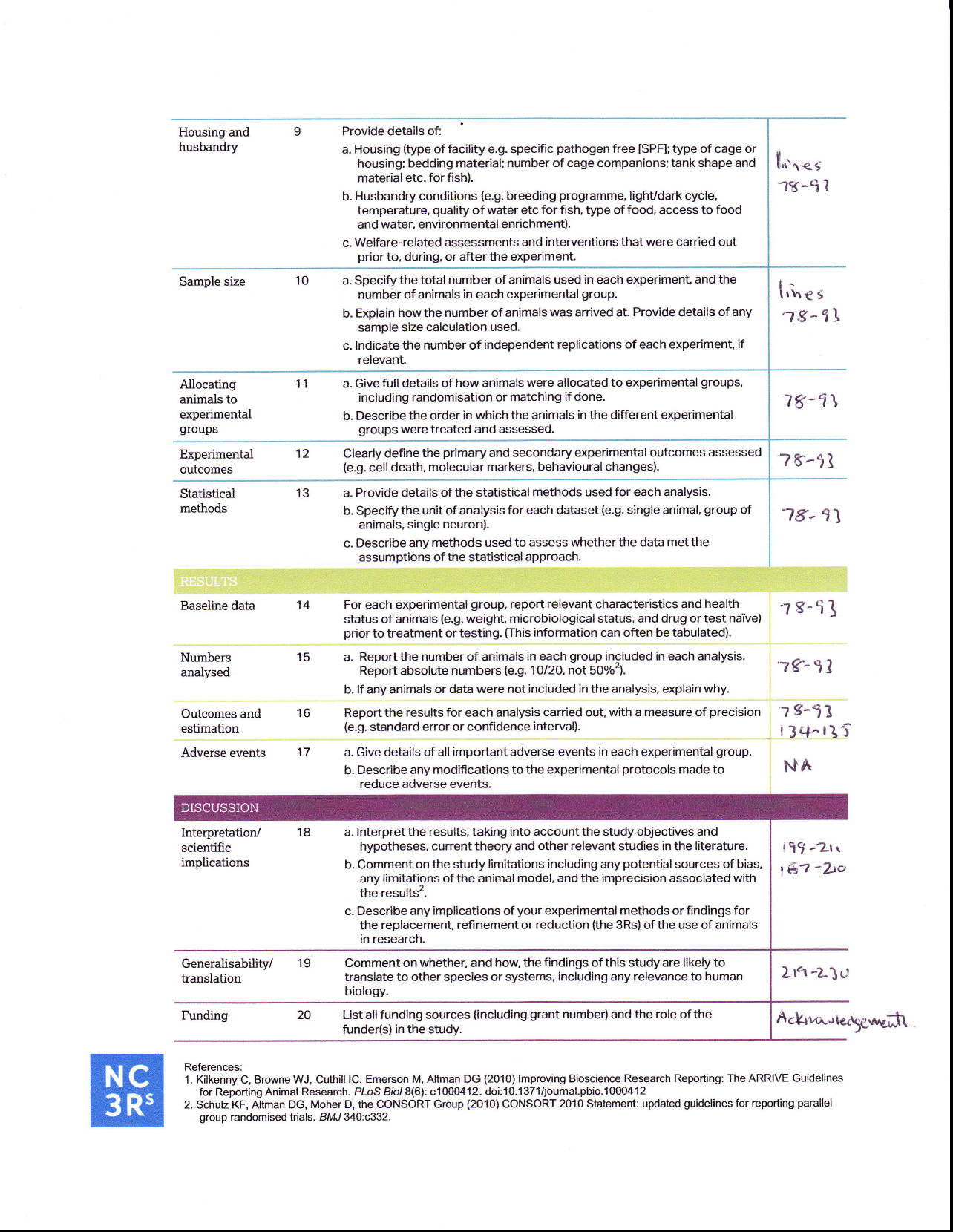

Supplement: S1 Text — (DOCX) [file pone.0141407.s001.docx]
